# Excretion and Residual Concentration Correlations of Salbutamol Between Edible Tissues and Living Samples in Pigs and Goats

**DOI:** 10.3389/fphar.2021.754876

**Published:** 2021-11-25

**Authors:** Lei Sun, Minjuan Zhu, Jingfei Shi, Kun Mi, Wenjing Ma, Xiangyue Xu, Hanyu Wang, Yuanhu Pan, Yanfei Tao, Zhenli Liu, Lingli Huang

**Affiliations:** ^1^ MOA Laboratory of Risk Assessment for Quality and Safety of Livestock and Poultry Products, Wuhan, China; ^2^ National Reference Laboratory of Veterinary Drug Residues (HZAU) and MAO Key Laboratory for Detection of Veterinary Drug Residues, Wuhan, China; ^3^ College of Veterinary Medicine of Huazhong Agricultural University, Wuhan, China

**Keywords:** SAL, pig, goat, excretion, depletion, pre-slaughter monitoring

## Abstract

Illegal use of salbutamol (SAL), a β-adrenergic leanness-enhancing agent, has posed potential threat to human health in China. The excretion and depletion of SAL in pigs and goats were investigated, and the concentration correlations between edible tissues and living samples were analyzed to find out a suitable living sample for pre-slaughter monitoring of SAL in pigs and goats. After a single oral dosage of 1.2 mg/kg SAL, approximately 70% of the dose was excreted by pigs and goats from their excreta. When pigs and goats were supplied feed containing SAL (20 mg/kg) for 14 consecutive days, high concentrations of SAL were observed in the liver and kidneys, and the longest persistence was observed in hair. Unlike pigs, SAL was presented primarily as conjugated SAL in goats. Excellent concentration correlations of SAL were observed between urine and edible tissues both in pigs and goats, and in addition, good correlations also were found between hair and edible tissues in pigs and between feces and edible tissues in goats. Hence, urine and hair could accurately predict SAL concentrations in edible tissues of pigs, whereas feces and urine were satisfactory for predicting SAL concentrations in edible tissues of goats. These data make it possible for pre-slaughter monitoring of SAL residues in the edible tissues of pigs and goats.

## Introduction

β-agonists, a class of drugs with the structure of β-phenylethanolamine, have the effects of relaxing smooth muscles and releasing bronchospasm and have been the first choice for treating asthma and chronic obstructive pulmonary disease (COPD) (Petta et al., 2017; Price et al., 2018). It had been applied in livestock to promote animal growth and increase fat catabolism and lean meat rate in the early 1980s (Sung et al., 2015; Montes Nino et al., 2017). However, they would cause diseases of the human cardiovascular and nervous system if they were consumed by the people through animal products (Cheng et al., 2013; Mi et al., 2014). Salbutamol (SAL), as one of the β-agonists, has been banned from livestock in many countries, including China (Li et al., 2015). However, SAL is still illegally used in pigs and goats in China to improve carcass composition and gain high economic benefits. Since β-adrenoceptor agonists are potentially harmful to human health and of great concern worldwide for its safety, the illegal use of SAL in food given to animals must be monitored, and its residues in animal production should be predicted before its application in the market.

Liquid chromatography–tandem mass spectrometry (LC-MS/MS) has become the mainstream method for monitoring SAL residues because of its high sensitivity and selectivity compared with biological methods, such as enzyme-linked immunosorbent assay (ELISA) (Li et al., 2015; Xiong et al., 2015; Montes Nino et al., 2017). The traditional monitoring of SAL residues in edible tissues requires to slaughter the animals and detect abundant tissue samples (Cao et al., 2013; Zhang et al., 2017b; Yang et al., 2018), which is time- and labor-consuming. Furthermore, once the residue concentration is higher than the maximum residue limit (MRL), the edible tissue must be destroyed which would cause huge economic losses. As early as the 1970s, the concept of using living samples to predict drug residues in edible tissues had been proposed (Nouws and Ziv, 1978). Living samples, such as biological fluids (plasma, urine), feces, and hair, can be easily and readily obtained without slaughtering animals. Then, by establishing the correlations of drug concentration between edible tissues and living samples, the real-time residue concentration of the drug in the corresponding tissue of each animal can be predicted. Some reports had demonstrated that the concentration of ractopamine, a representative of β-adrenoceptor agonist, in urine, plasma, and hair was significantly correlated with the concentrations in the edible tissues of pigs and goats (Huang et al., 2016; Aroeira et al., 2019). These studies confirmed that living samples would be an efficient and economic strategy to monitor the illegal use of SAL as feed additive in livestock.

The dynamic process of SAL in several animals had been studied in the past years. The excretion of SAL had been well-investigated in humans, cow, sheep, and mouse (Vulić et al., 2011; Dickinson et al., 2014; Chakrabarty et al., 2020), but not in pigs and goats. Several papers reported SAL could be accumulated in the liver and hair of mouse (Vulić et al., 2011; Vulić et al., 2014). Zhang et al. (2016) and Zhang et al. (2017b) revealed the elimination of SAL in hair, plasma, urine, and edible tissues of cattle. Yang et al. (2018) reported the depletion of SAL in hair, plasma, urine, and edible tissues of goats and chickens. However, the depletion of SAL in pigs remains unclear, and no studies focused on the residual concentration correlation of SAL between the edible tissue and living samples in any food animals.

The present study investigated the depletion and excretion of SAL in pigs and goats using ultra-performance liquid chromatography–tandem mass spectrometry method and established the concentration correlations of SAL between edible tissues and living samples (plasma/urine/hair/feces). Based on these correlations, the optimal living sample was identified as a representative matrix for monitoring the illegal use of SAL in food animals. These results may provide a feasible pre-slaughter living monitoring method to estimate the concentration of SAL in the edible tissues of pigs and goats before the animal products are exposed to consumers.

## Materials and Methods

### Reagents and Materials

The standard of SAL sulfate (≥99.5%) was obtained from Sigma-Aldrich Chemie b.v. (Zwijndrecht, Netherlands), β-glucuronidase was purchased from Sigma Chemical Co. (Helix pomatia, Type H-2, aqueous solution, ≥85,000 units/ml). High-performance liquid chromatography (HPLC)–grade methanol was purchased from Merck Chemicals Co. (Darmstadt, Germany). Ultrapure water was supplied by (Milli-Q; Millipore, Bedford, MA, United States). Formic acid, ammonia, N-hexane, ammonium acetate, and ethyl acetate were all of analytical grade and purchased from Shanghai Guoyao Company (Shanghai, China). Oasis MCX solid-phase extraction cartridges were purchased from Waters Co. (Milford, MA, United States).

### Solutions

The standard stock solution (1.0 mg/ml) was prepared in methanol and stored at −20°C for 3 months. The standard working solution (100 μg/ml) was prepared weekly by diluting the stock solution with methanol. Ammonium acetate solution (0.2 N) was prepared as follows: 15.4 g ammonium acetate was dissolved in water in a beaker, then the solution was transferred to a 1,000-ml volumetric flask and water was added to the mark. Acetic acid was used to adjust the pH to 5.2. Ammoniated ethyl acetate solution (5%) was prepared by mixing 25 ml of ammonia and 500 ml of ethyl acetate. Aqueous formic acid (2%) was prepared by adding 22.4 ml of formic acid in a 1,000-ml volumetric flask and then diluting to the mark with water. Mobile phase A (0.1% formic acid + 5 mmol ammonium acetate) was prepared as follows: 0.38 g ammonium acetate was dissolved in water in a beaker. Then, 1 ml of formic acid was added. The mixture was transferred to a 1,000-ml volumetric flask and water was added to the mark. Mobile phase B was HPLC-grade methanol.

### Animals

The use of animals in this study was allowed in accordance with the Guidelines of the Committee on the Care and Use of Laboratory Animals of China (permit SYXK 2007-0044). The studies were conducted on 29 healthy Landrace–Large White crossbred castrated male pigs (weight, 25 ± 2 kg) and Boer goats (weight, 20 ± 2 kg). The pigs were purchased from the China Breeding Swine Testing Center (Wuhan, China) and the goats were purchased from Huazhong Agricultural University Veterinary Hospital (Wuhan, China). Before the formal experiment began, all animals were acclimatized for 7 days. A standard ration based on corn and soybean was fed twice a day, and tap water was available *ad libitum*. The animals were starved for 6 hours before dosing.

### Experimental Design

Pigs were randomly divided into groups A, B, and C. Group A (*n* = 5) was the control group which provided standard ration (without SAL). Group B (*n* = 4) was the metabolic group which administrated SAL by oral gavage singly at a dose of 1.2 mg/kg bw. Group C (*n* = 20) was the residual group which provided specific feed containing SAL at a dose of 20 mg/kg for 14 consecutive days. For group B, urine and feces were collected at 0–6, 6–12, and 12–24 h and every 24 h thereafter, until SAL cannot be detected in the collected samples. All urine and feces samples were weighed and stored at −20°C. A group of pigs (*n* = 5) was slaughtered at withdrawal time of 0.25, 1, 3, 7, and 14 days, including one in group A and four in group B. The methods of slaughtering and bloodletting were in compliance with the guidelines provided by the American Veterinary Medical Association (2001). Edible tissues (liver, kidneys, muscles, fat, lungs, large intestine, and small intestine) and living samples (plasma, urine, feces, and hair) were collected. All samples were frozen at −20°C until analysis. The experimental design of goats was the same as that of pigs.

### Sample Extraction and Purification

The extraction and purification of samples were based on the method of Huang et al.^15^ which is briefly described as follows. When the sample was not required to be hydrolyzed, β-glucuronidase and incubation steps should be omitted.

#### Plasma

Plasma (2 ml) was placed in a 50-ml disposable plastic centrifuge tube. 3 ml of acetate buffer (0.2 mol/l, pH = 5.2) and 40 μl β-glucuronidase were added. Then, the mixture was incubated at 37°C for 12 h. 4 ml of methanol was added. Then, the mixture was centrifuged at 8,000 rpm for 10 min, and the supernatant was collected for further use.

#### Urine

Urine (2 ml) was placed in a 50-ml disposable plastic centrifuge tube. 3 ml of acetate buffer (0.2 mol/l, pH = 5.2) and 40 μl β-glucuronidase were added. Then, the mixture was incubated at 37°C for 12 h. 6 ml of ammonia-ethyl acetate (5%) was added to the mixture. After the mixture was centrifuged at 8,000 rpm for 10 min, the supernatant was transferred to a new tube. The residue was extracted twice with 6 ml of ammonia-ethyl acetate (5%), and three times the extracts were combined with the supernatant and 4 ml of formic acid (2%) was added. After the mixture was evaporated to dryness under a nitrogen stream at 45°C, 2 ml of n-hexane was added. Then, the mixture was centrifuged at 8,000 rpm for 10 min, and the supernatant was collected for further use.

#### Hair

1.0 g of hair was immersed in 40 ml Tween 80 solution (0.2%) for 30 min and mixed in an ultrasonic bath for 20 min. After the hair was rinsed with distilled water and dried in a drying cabinet. 500 mg of dried hair was cut into small pieces and 4 ml of NaOH (1 M) was added. Then, the mixture was heated in a 65-°C water bath for 2.5 h. 3.5 ml of HCl (1 M) and 5 ml of ethyl acetate were added, and the extracted solution was transferred to a new tube. Then, repeated extraction was conducted with ethyl acetate. The combined extracts were centrifuged at 8,000 rpm for 10 min, the supernatant was transferred to a new tube, and 4 ml of formic acid (2%) was added. After the mixture was evaporated to dryness under a nitrogen stream at 45°C, 2 ml of n-hexane was added. Then, the mixture was centrifuged at 8,000 rpm for 10 min and the collected solution was taken out for further use.

#### Tissues and Feces

2.0 g of tissue or feces was added into a 50-ml disposable plastic centrifuge tube, and 3 ml of acetate buffer (0.2 mol/l, pH = 5.2) and 40 μl β-glucuronidase were added. Then, the mixture was incubated at 37°C for 12 h. 6 ml of ammoniated ethyl acetate (5%) was added to the mixture, and after the mixture was centrifuged at 8,000 rpm for 10 min, the supernatant was transferred to a new tube. The residue was extracted twice with 6 ml of ammoniated ethyl acetate (5%) and three times the extracts were combined with the supernatant and 4 ml of formic acid (2%) was added, After the mixture was evaporated to dryness under a nitrogen stream at 45°C, 2 ml of n-hexane was added. Then, the mixture was centrifuged at 8,000 rpm for 10 min and the collected solution was taken out for further use.

#### Sample Purification

The extracted solution was loaded onto the activated MCX cartridge column (60 mg, 3 ml) (Waters Corp., Milford, MA, United States). The column was washed with formic acid (2%) and methanol successively. A mixture of methanol and ammonia (20:1) was used for elution. The eluent was evaporated to dryness under a nitrogen stream at 40°C, and 1 ml of the initial mobile phase was used for reconstitution.

### LC-MS/MS Analysis

Chromatographic separation was carried out on a Finnigan Surveyor HPLC system (Thermo Fisher Scientific, United States) with a Thermo Hypesil Gold C18 (150 mm × 2.1 mm, 5 μm) column at 40°C, which included an online degasser and a Surveyor autosampler. The flow rate was set at 0.25 ml/min. The injection volume was 10 μl. Mobile phase A was methanol, whereas mobile phase B was 0.1% formic acid solvent containing 5 mmol/l ammonium acetate. Using gradient elution, the specific settings are set as follows: 0.0–1.0 min, A/B (10/90); 9.0–15 min, A/B (90/10); and 16.0–22.0 min, A/B (10/95). It is necessary to rinse the needle with H_2_O/methanol (50/50, v/v) for 8 min between injections to ensure the accuracy of quantitative results. Mass spectrometric acquisition was performed on a Micromass-Quattro Premier XE mass spectrometer (Waters, Milford MA, United States) using the positive electrospray ionisation mode (ESI+). The capillary voltage and spray voltage were maintained at 3.5 and 4.6 kV, respectively. The sheath and aux gas pressures were maintained at 30 and 10 arb, respectively. The capillary temperature was 350°C, and the tube lens offset was 104 V. The parent ion of SAL was 240.100 m/z. whereas the quantitative daughter ions of SAL were 121.082, 148.060, and 222.163 m/z and the collision energies (Ev) of the daughter ions were 28, 16, and 8 eV.

### Data Analysis

Mean, SD, and CV were calculated in Excel. The half-life (t_1/2_) of SAL in edible tissues and living samples were calculated graphically by fitting linear regression. Linear regression, exponential function, and power function were used to describe the concentration correlations of SAL between edible tissues and living samples in pigs and goats by using SPSS software. All bar graphs were generated using GraphPad Prism 5.0 (GraphPad Software, La Jolla, CA, United States). Statistical analysis was performed with *p*-test using SPSS software. The *p-value* < 0.05 was considered to indicate statistical significance.

## Results

### Method Validation

The developed method was validated according to the validation guideline supplied by the EU Commission Decision 2002/657/EC.25 (2002). The verification objects included specificity, matrix effects, linear relationship, CCα CCβ, accuracy, and precision. The specificity refers to the distinction between the analyte and impurities. By comparing the chromatogram of the blank matrix extract solution with that of spiked matrix extract solution, we inferred that there was no interference around the SAL retention time. Blank samples of pigs and goats were used as matrices for calibration curve study and the matrix-matched calibration curves were assessed at seven levels as follows: 0.5, 1.0, 2.0, 4.0, 8.0, 16, and 32 μg/kg (*n* = 3). Regression coefficients (r) were higher than 0.999 in all matrixes. CCα values were defined as three times the signal−noise ratio (S/N) and established by the following steps: 20 blank samples of pigs and goats were analyzed, and the S/N was calculated at the time window in which the analyte was expected. CCβ was calculated by analyzing 20 blank samples spiked with the concentrations at CCα, and the CCα value plus 1.64 times the corresponding standard deviation (SD) was equal to CCβ (β = 5%). The CCα and CCβ values of the method were within the range of 0.34–0.41 and 0.44–0.57 μg/kg in the tissues of pigs and goats, respectively, but 1.1 and 1.2 μg/kg in hair of pigs and goats, respectively. Accuracy and precision are expressed in terms of recovery and relative standard deviation (RSD), respectively. The recovery and RSD were determined by analyzing the blank sample–fortified SAL standard solution at the levels of 0.25, 0.5, and 1.0 μg/kg, respectively. Each concentration was measured in five parallel sessions for three consecutive days. The recovery rate in the edible tissues of pigs and goats ranged within 74.6–91.3% and the RSD was less than 10.8%. The recovery rate in living samples (plasma/urine/hair/feces) of pigs and goats ranged within 80.0–89.2% and the RSD was less than 11.1%.

### Excretion of SAL in Pigs and Goats

**FIGURE 1:**
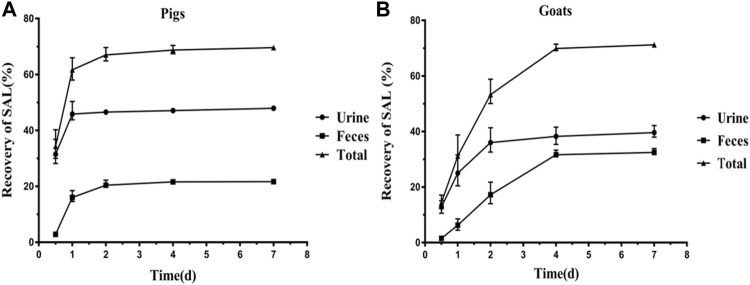
Excretion of SAL (SAL) in pigs **(A)** and goats **(B)** after a single oral administration at 1.2 mg/kg bw (mean ± SD).

**FIGURE 2:**
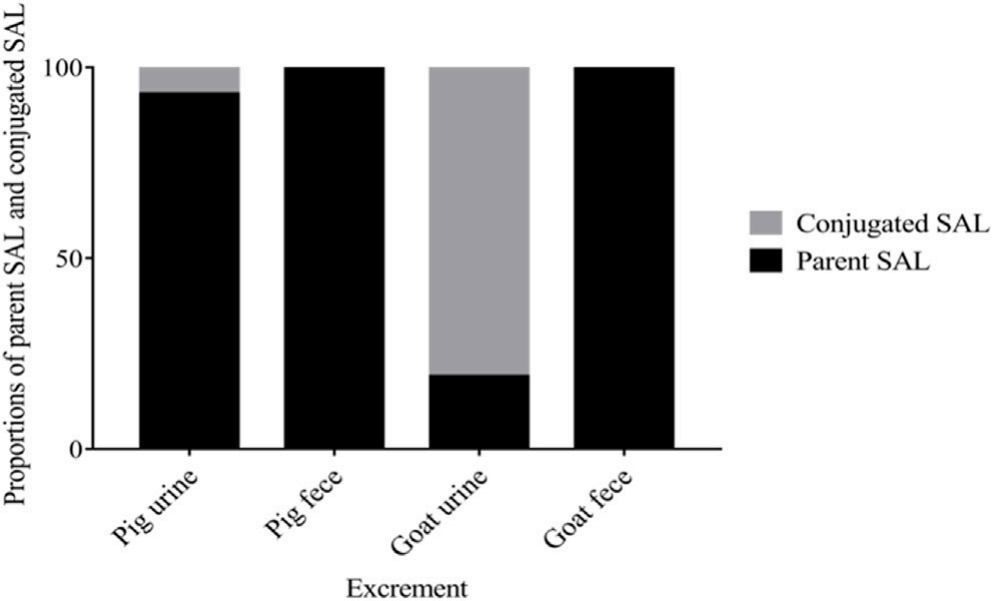
Proportions of parent SAL and conjugated SAL in urine and feces of pigs and goats throughout the trial.

The excretion data of SAL in pigs and goats are summarized in Figures 1, 2. Within 6 days after a single oral dosage of 1.2 mg/kg SAL, 69.62% of the dose was excreted by the pigs, of which 47.94% was recovered in urine and 21.68% was recovered in feces. In goats, a total of 71.22% of the dose was excreted, and its recovery in urine and feces was 39.64 and 32.47%, respectively. The excretion rate of SAL in pigs was about twice that of goats, because at withdrawal times of 1 d, the total recovery rate of SAL in pigs was 61.56%, while that of goats was only 31.32%. In addition, SAL was present as parent SAL in feces of pigs and goats, but conjugated SAL was detected in urine of pigs and goats. Notably, the proportion of conjugated SAL in urine of goats (81%) was obviously higher than that of pigs (3%).

### Depletion of SAL in Pigs and Goats

**FIGURE 3:**
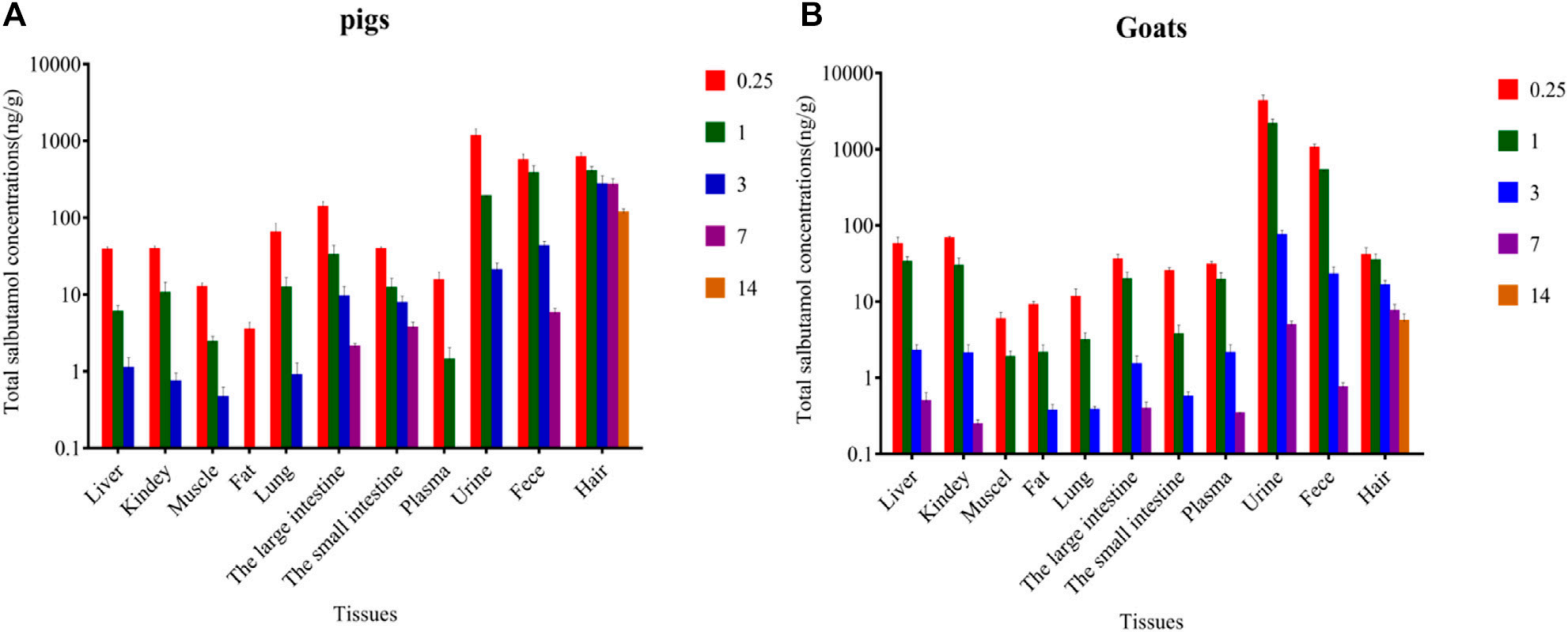
Total SAL concentrations in various tissues of pig **(A)** and goat **(B)** at withdrawal times of 0.25, 1, 3, 7, and 14 days, respectively (mean ± SD).

Total SAL concentrations in edible tissues and living samples (plasma/urine/hair/feces) of pigs and goats at different withdrawal times are shown in Figure 3. The residual concentrations of total SAL in goat edible tissue were generally very low, but those in the liver (55.08 μg/kg), kidneys (65.49 μg/kg), and fat (8.73 μg/kg) were about two times than those in pig. At withdrawal times of 0.25 days, the peak concentrations of total SAL in edible tissues were found in the large intestine (131.3 μg/kg) of pigs and kidney (65.49 μg/kg) of goats. Total SAL remained in most edible tissues of pigs and goats for more than 3 days, but was undetectable only after the withdrawal time of 1 day in the fat and plasma of pigs and the muscles of goats. Total SAL concentrations in plasma, urine, and feces of goats were all significantly higher than those in pigs (*p* < 0.05), especially in urine because total SAL concentration in goat’s urine (4,149 μg/kg) was approximately four times than that in pigs (1,120 μg/kg). The longest persistence of SAL was observed in hair of pig and goat for 14 days, and at withdrawal time of 14 days, total SAL concentration in pig’s hair (57.26 μg/kg) was 10 times than that in goat’s (5.43 μg/kg).

**FIGURE 4:**
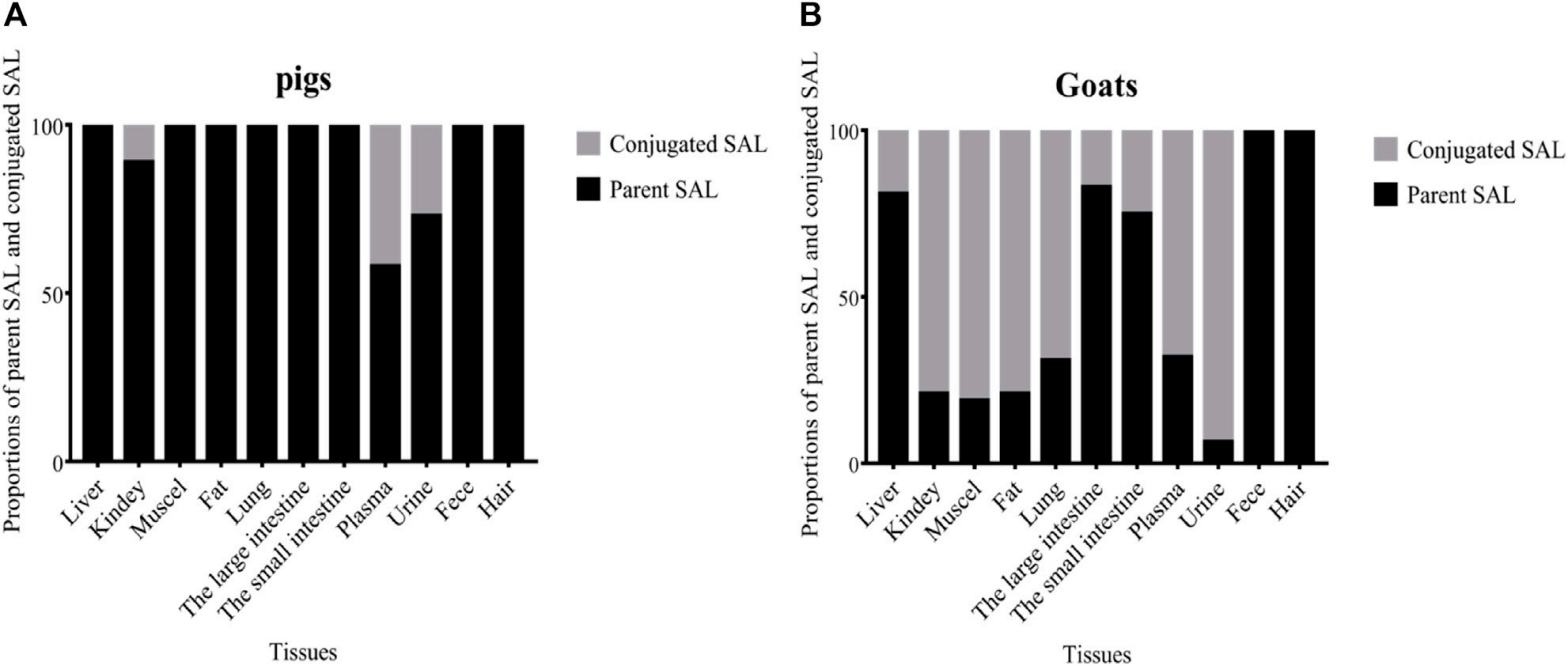
Proportions of parent SAL and conjugated SAL in various tissues of pig **(A)** and goat **(B)** at withdrawal times of 0.25 days.

Proportions of parent SAL and conjugated SAL in edible tissues and living samples (plasma/urine/hair/feces) of pigs and goats at withdrawal time of 6 h are shown in Figure 4. SAL mainly presented as parent SAL in pigs’ edible tissues. Conjugated SAL was only detected in the kidneys of pigs and the proportion was only 11%, which was significantly lower than that of parent SAL (89%) (*p* < 0.01). The opposite scenario holds true in goats’ edible tissues, because conjugated SAL was detected in all edible tissues of goats, and the proportions in kidneys, muscles, fat, and lungs were all up to 70%, whereas small amount of conjugated SAL was detected in the liver, large intestine, and small intestine of goats and the proportions were less than 25%. Conjugated SAL was also detected in plasma and urine of pigs and goats. Notably, the proportions in goat’s plasma (68%) and urine (93%) were extremely higher than those in pig’s, which was 42 and 27%, respectively. Only parent SAL was detected in feces and hair of pigs and goats.

The elimination parameters were calculated by drawing the elimination curve of total SAL in edible tissues and living samples of pigs and goats (Table 1). SAL depleted too quickly in fat and plasma of pigs and muscle of goats to draw their elimination curve. Whereas SAL had the longest elimination half-life (t_1/2_) in hair of pigs and goats and the t_1/2_ was 5.07 and 7.6 days, respectively. The t_1/2_ in the kidney and lungs of pigs and goats was less than 0.5 days. SAL depleted more rapidly in the intestines of goats with t_1/2_ ranging within 0.54–1.14 days compared with that of pigs with t_1/2_ ranging within 1.55–3.51 days.

**TABLE 1:** Elimination parameters of SAL in edible tissues, plasma, and hair of pigs and goats.

Samples	Elimination equation	Elimination rate constant (day^−1^)	T_1/2_ (day)
**Pigs**			
Liver	C = 33.37e^−1.19t^	1.19	0.58
Kidney	C = 41.68e^−1.14t^	1.14	0.61
Muscle	C = 11.46e^−1.12t^	1.12	0.62
Lung	C = 76.57e^−1.52t^	1.52	0.45
Large intestine	C = 43.31e^−0.45t^	0.45	1.55
Small intestine	C = 14.18e^−0.20t^	0.2	3.51
Hair	C = 378.42e^−0.14t^	0.14	5.07
**Goats**			
Liver	C = 35.86e^−0.66t^	0.66	1.06
Kidney	C = 95.51e^−1.28t^	1.28	0.54
Fat	C = 8.85e^−1.09t^	1.09	0.63
Lung	C = 12.74e^−1.20t^	1.2	0.58
Large intestine	C = 20.49e^−0.61t^	0.61	1.14
Small intestine	C = 22.68e^−1.29t^	1.29	0.54
Plasma	C = 24.56e^−0.64t^	0.64	1.08
Hair	C = 17.78e^−0.09t^	0.09	7.6

### Concentration Correlations of Total Sal Between Edible Tissues and Living Samples in Pigs and Goats

**FIGURE 5:**
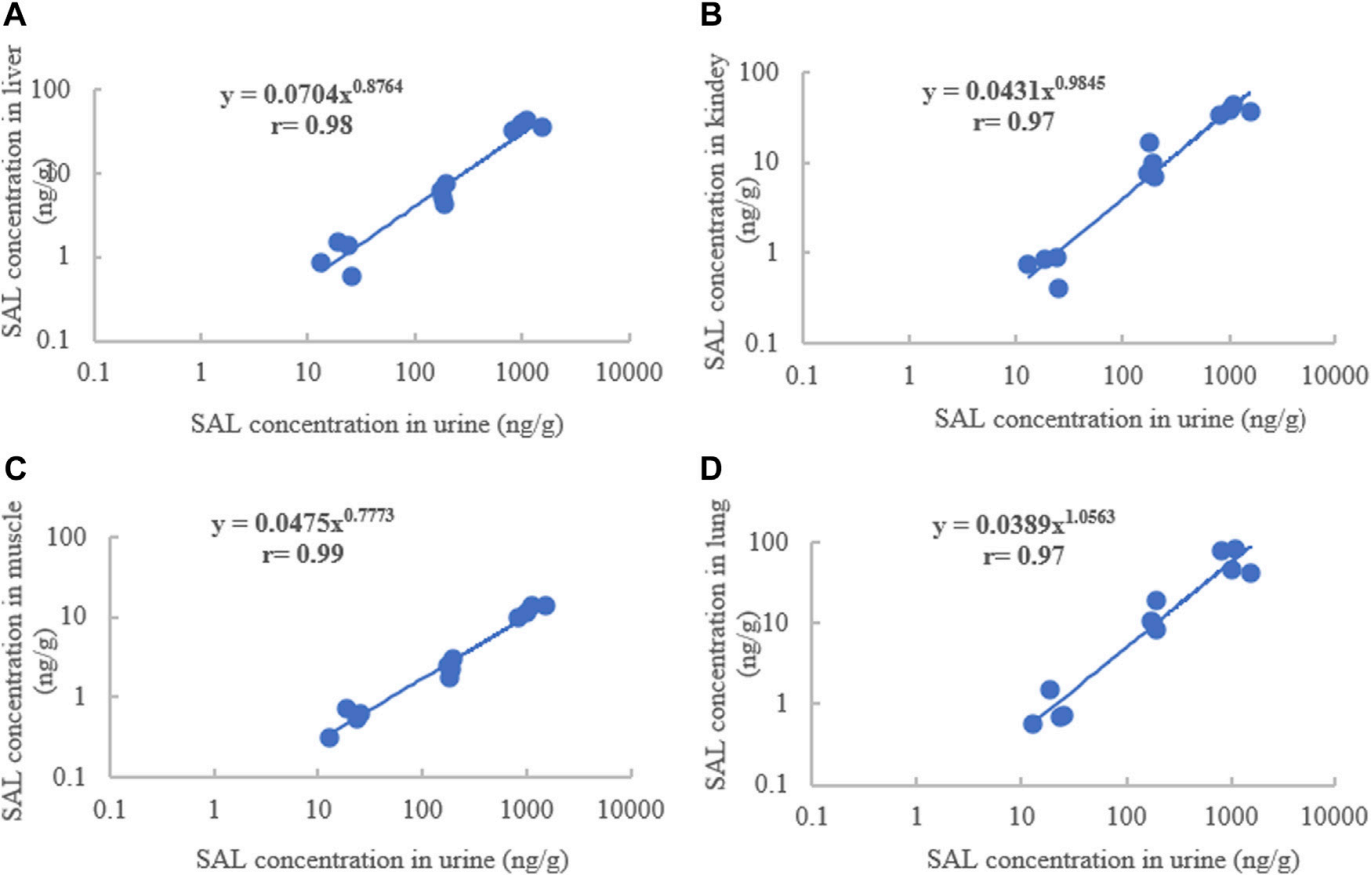
Concentration correlations of SAL between urine and the liver **(A)**, kidney **(B)**, muscles **(C)**, and lungs **(D)** of pigs.

The exponential function, power function, and linear regression were used to describe the concentration correlations of total SAL between edible tissues and living samples in pigs and goats. In pigs, due to SAL being rapidly depleted in plasma and only detected at the first two time points of withdrawal, the correlations of total SAL concentration between plasma and edible tissues could not be performed. Regardless of which functions were used to establish the correlation, no good concentration correlation of total SAL was found between hair/feces and edible tissues. However splendid correlations of total SAL concentration were established between urine and the liver, kidneys, muscles, and lung when using power function for correlation description (Figure 5). The correlation coefficients ranged from 0.97 to 0.99. The highest correlation coefficient (0.99) between urine and muscles was obviously superior to that between urine and other edible tissues.

Concentration correlations of total SAL between edible tissues and living samples in goats are summarized in Table 2. Excellent concentration correlations of total SAL were established between feces and all edible tissues, but except in muscles because SAL was undetectable only at the withdrawal time of 3 days. The correlation coefficients matched by the power function were 0.98, 0.99, and 0.97 between feces and the liver, kidneys, and large intestine, respectively, and those matched by exponential function were 0.96, 0.98, 0.98 between feces and fat, lungs, small intestine, and respectively. Urine could accurately predict total SAL concentrations in all edible tissues (except muscles), when the relationships of the total SAL concentration between urine and fat and small intestine were described by exponential function, and the correlation coefficients were 0.96 and 0.95, respectively. When power function was used to establish the concentration correlations of total SAL between urine and the liver, kidneys, lungs, and large intestine, the correlation coefficients were 0.99, 0.99, 0.96, and 0.98, respectively. Satisfactory correlations were also found between plasma and most edible tissues (liver, kidneys, lungs, and large intestine), and a correlation coefficient of 0.97 between plasma and lungs was matched by exponential function. While power function indicated strong correlation correlations between plasma and the liver, kidneys, and large intestine, the correlation coefficients were all above 0.99.

**TABLE 2:** Residual concentration correlations of total SAL between edible tissues and living samples in goats.

Living samples-edible tissues	Regression equation	Correlation coefficient (r)
Feces-liver	y = 0.4528x^0.6666^	0.98
Feces-kidney	y = 0.2558x^0.7663^	0.99
Feces-fat	y = 0.3698e^0.0031x^	0.96
Feces-fat	y = 0.3698e^0.0031x^	0.96
Feces-large intestine	y = 0.3462x^0.631^	0.97
Feces-small intestine	y = 0.5211e^0.0037x^	0.98
Urine-liver	y = 0.1335x^0.7112^	0.99
Urine-kidney	y = 0.063x^0.817^	0.99
Urine-fat	y = 0.3979e^0.0007x^	0.96
Urine-lung	y = 0.0127x^0.7687^	0.96
Urine-large intestine	y = 0.1071x^0.6763^	0.98
Urine-small intestine	y = 0.5959e^0.0009x^	0.95
Plasma-liver	y = 1.325x^1.0732^	0.99
Plasma-kidney	y = 0.8862x^1.228^	0.99
Plasma-lung	y = 0.3192e^0.1162x^	0.97
Plasma-large intestine	y = 0.9542x^1.0177^	0.99

## Discussion

### Excretion of SAL in Pigs and Goats

Existing data on SAL excretion in dogs, rats, rabbits, and humans indicated that when administered orally, SAL could be excreted rapidly and urine was the primary excretion route (Mareck et al., 2011; Vulić et al., 2011; Dickinson et al., 2014; Chakrabarty et al., 2020). In the present study, SAL excretion in pigs and goats was first revealed. Within 6 days after a single oral dosage of 1.2 mg/kg SAL, 69.62% of the dose was excreted by the pigs, and the recovery rate in urine (47.97%) was twice that in pigs feces (21.68%), which further confirmed that SAL was excreted mainly via urine when administered orally, while in goats a total of 71.22% of the dose was excreted, of which 39.64% was excreted via urine and 32.47% was excreted via feces, although the recovery rate in urine was higher than that in feces, but feces as an important excretion route cannot be ignored in goats. Huang et al. reported that the excretion rate of ractopamine in pigs was about twice that of goats (Huang et al., 2016), while in our study, SAL, which as a congener of ractopamine, also exhibited this phenomenon. At withdrawal time of 1 day, the recovery rate of SAL in pigs was up to 61.56%, but that of goats was only 31.32%. This can be interpreted as the ruminant’s physiological structure being different from that of monogastric animals and rumen’s unique function makes the excretion of SAL in goats more complicated.^24^ Several studies have reported that glucuronide-SAL is the main metabolite of SAL in cow, pig, rat, and human (Mareck et al., 2011; Domínguez-Romero et al., 2013; Wu et al., 2013; Zhang et al., 2017b). Thus, all samples were hydrolyzed with β-glucuronidase. Our results have shown that 81% of the excreted drugs in urine of goats were glucuronide-SAL, which was significantly higher than that in urine of pigs (3%), which means that SAL could conjugate with glucuronide more easily in goats’ urine than in pigs’ urine.

### Depletion of SAL in Pigs and Goats

Consuming animal products contaminated with β_2_ agonists will lead to food poisoning (Wu et al., 2013; Bovee et al., 2016; Hieger et al., 2016). Therefore, SAL residues in edible tissues must be strictly monitored. Studies on the depletion of SAL in cattle, goats, chickens, and mice clarified that SAL tends to accumulate in the liver and kidneys and that the residues are seldom found in muscles and fat (Cao et al., 2013; Vulić et al., 2014; Zhang et al., 2017b; Yang et al., 2018). In the present study, high concentrations of total SAL were, indeed, observed in the liver and kidneys of pigs and goats, and concentrations of SAL in goats’ liver and kidneys (55.08, 65.49 μg/kg) were about twice those of pigs (37.49, 37.98 μg/kg). But, the residual concentrations of total SAL in muscles and fat was relatively low at less than 13 μg/kg. These observations indicated that the liver and kidneys were more suitable as targets tissues for residue monitoring of SAL. High concentrations of total SAL were also found in large and small intestines, especially in pigs (133.8, 37.32 μg/kg). The results remind that researchers should focus on SAL residues in the intestines because they are a common food in many countries, including China. As a short-acting β_2_-agonist, the concentration of SAL observed in the lungs was generally low (Zhang et al., 2017b; Yang et al., 2018). In our research, the total SAL concentration in the lungs of goats was only 11.23 μg/kg, which is consistent with previous reports, but that in pigs was unexpectedly high (67.24 μg/kg). In the present study, the residual concentrations were higher than those in the study of Hung et al., which may be due to the different sampling time and dosage. To a certain extent, it is also affected by enzymatic hydrolysis. Total SAL concentration was rapidly depleted in fat and muscles and was undetectable at a withdrawal time of 7 days. By analyzing the proportions of parent SAL and conjugated SAL in edible tissues, we discovered that SAL mainly presented as parent SAL in pig’s edible tissues, while the opposite scenario holds true in goat’s edible tissues. This difference indicated that SAL could conjugate with glucuronide more easily in goats’ edible tissues than in pigs’ edible tissues.

Total SAL concentration in urine was significantly higher than that in plasma (*p* < 0.01), whether in pigs or goats. This is basically consistent with the reports of SAL in cattle, horses, and humans (Wieder et al., 2015; Zhang et al., 2016; Zhang et al., 2017b; Ye et al., 2019). Moreover, at the withdrawal time of 0.25 days, total SAL concentration in goat’s urine and plasma was 4 and 2 times that in pig’s urine and plasma, respectively, and the proportions of conjugated SAL in goat’s urine and plasma were also significantly higher than those in pigs. These data indicated that SAL could accumulate and conjugate with glucuronide more easily in goat’s urine and plasma than in pigs. Our study first reported the depletion of SAL in feces. Feces as the secondary excretion route of SAL in pigs and goats also had high residual concentration of total SAL, and the total SAL concentration in goat’s feces (1,016 μg/kg) was twice than that in pig’s (541.9 μg/kg) at the withdrawal time of 0.25 days. Only parent SAL was detected in feces. Several studies have reported that melanin has a special adsorption effect on SAL (Howells et al., 1994; Hu et al., 2000). This leads to the accumulation of SAL in animal hair and retina. Considering that removing the eyes of live animals is cruel and harmful to animal health, we gave up using the eye as a living sample. Different from urine, plasma, and feces, the concentration of total SAL in pig’s hair (596.5 μg/kg) was 14 times that in goats, which means that SAL was more likely to accumulate in pig’s hair than in goat’s hair. However, SAL persisted in hair of goats as long as in hair of pigs, and only parent SAL was detected in hair of pigs and goats.

### Concentration Correlations of Total SAL Between Edible Tissues and Living Samples in Pigs and Goats

The concentration correlations of total SAL between edible tissues and living samples of pigs and goats were established for the first time in our study. Previous studies have shown that SAL is present primarily as a mixture of parent SAL and glucuronide-SAL (Mareck et al., 2011; Domínguez-Romero et al., 2013; Wu et al., 2013; Zhang et al., 2017b). Thus, total SAL is more suitable as a marker residue than parent SAL. Due to high accumulation and longest persistence of SAL in hair, many scholars have proposed that hair can be used as a vital indicator to monitor the illegal use of SAL (Zhang et al., 2016; Zhang et al., 2017a; Liu et al., 2019). In our study, unfortunately, regardless of which functions were used to establish the correlation, no good correlations of total SAL concentration were found between hair and edible tissues of pigs and goats.

Plasma and urine are considered ideal indicators for monitoring drug residues in edible tissues (Liu et al., 2010; Chiesa et al., 2012; Aroeira et al., 2019). However, SAL was rapidly depleted in plasma and was only detected at the first two time points of withdrawal, so the correlations of total SAL concentration between plasma and edible tissues of pigs could not be performed. Excellent correlations of SAL concentration between plasma and most tissues of goats were established, which indicated that plasma could be used to monitor SAL residues in edible tissues of goats. Urine was a satisfactory indicator for predicting SAL residues in edible tissues of pigs and goats because outstanding correlations of SAL concentrations between urine and most edible tissues of pigs and all edible tissues of goats were observed in this study.

Based on the results of correlation analysis, the purpose of pre-slaughter living monitoring the residues of SAL in edible tissues of pigs and goats was achieved. The pre-slaughter living monitoring strategy could efficiently, quickly, and accurately monitor the residues of SAL in edible tissues of pigs and goats, thereby avoiding the economic losses caused by unnecessary slaughter and omissions caused by individual differences.

## Conclusion

A multiple residue detection method of SAL in 11 tissues, including liver, kidneys, muscles, and fat of pigs and goats, was established in this study, which would provide reliable techniques for the illegal use of SAL in pigs and goats. The excretion and the residue depletion of SAL in pigs and goats were first revealed. The excretion rate of SAL in pigs was higher than that in goats. SAL persisted the longest time in hair of both animals. Urine and hair were the residue prediction targets for SAL in pigs, while feces and hair were the prediction targets for SAL in goats. Urine and feces can be detected for drug residue in the main edible tissues. These findings provide a new *in vivo* predictive method for the residue monitoring of SAL in pigs and goats.

## Data Availability

The original contributions presented in the study are included in the article/Supplementary Material; further inquiries can be directed to the corresponding author.
