# Supplementary material for: Excretion and Residual Concentration Correlations of Salbutamol Between Edible Tissues and Living Samples in Pigs and Goats
Source: Front Pharmacol. 2021 Nov 25;12:754876. doi: 10.3389/fphar.2021.754876 (PMC8655863; doi:10.3389/fphar.2021.754876)
Supplement: Supplementary file 1 [file DataSheet1.PDF]

Table S1 CC_α and CC_β for tissues of swine

Tissue	CC_α(μg/L)	CC_β
Liver	0.39	0.53
Kidney	0.35	0.44
Muscle	0.39	0.52
Fat	0.38	0.51
Lung	0.36	0.48
Blood	0.34	0.43
Large intestine	0.35	0.45
Small intestine	0.36	0.47
Urine	0.35	0.46
Feces	0.38	0.52
Hair	1.08	1.16

Table S2 CC_α and CC_β for tissues of goats

Tissue	CC_α(μg/L)	CC_β(μg/L)
Liver	0.38	0.51
Kidney	0.35	0.44
Muscle	0.43	0.61
Fat	0.35	0.45
Lung	0.38	0.50
Blood	0.37	0.48
Large intestine	0.36	0.48
Small intestine	0.37	0.49
Urine	0.41	0.57
Feces	0.41	0.57
Hair	1.09	1.19

Table S3 The results of the stability

Standard stock solution		Standard working solution		Tissue with standard solution	
Time (month)	Concentration (μg/L)	Time (month)	Concentration (μg/L)	Time (month)	Concentration (μg/L)
0	1000	0	100	0	0.84±0.03
1	1007±2	1	99.5±2.9	1	0.85±0.03
3	997±1	2	98.4±0.9	2	0.83±0.01
6	994±1	3	95.5±1.5	3	0.83±0.02
12	990±1	4	92.1±2		

Table S4 The recoveries and variation of coefficients in spiked tissue of swine

Tissue	Concentration(μg/kg)	Recoveries (%)	variation of Coefficients (%)
Liver	0.25	81.3±8.4	10.4
	0.5	84.2±6.9	8.2
	1	86.1±7.4	8.6
Kidney	0.25	82.8±6.2	7.5
	0.5	84.6±5.5	6.4
	1	85.2±8.6	10.1
Muscle	0.25	84.2±8.3	9.8
	0.5	86.4±8.3	9.3
	1	91.3±10.2	11.2
Fat	0.25	78.7±7.9	10
	0.5	80.2±4.8	6
	1	81.1±6.6	8.1
Lung	0.25	86.5±7.0	8.1
	0.5	87.1±6.8	7.8
	1	86.2±6.6	7.7
Large intestine	0.25	83.0±6.1	7.5
	0.5	85.1±5.6	6.5
	1	84.9±6.6	7.7
Small intestine	0.25	84.9±6.8	8
	0.5	85.9±7.4	8.7
	1	85.1±8.5	9.9
Blood	0.25	83.4±5.6	6.7
	0.5	85.6±5.7	6.6
	1	88.3±7.5	8.5
Urine	0.25	80.0±6.4	8
	0.5	80.1±5.8	7.2
	1	82.0±7.6	9.3
Feces	0.25	80.3±8.2	10.2
	0.5	82.3±7.1	8.6
	1	82.6±6.3	6.5
Hair	0.25	64.6±4.8	7.4
	0.5	69.4±7.5	10.8
	1	76.8±7.1	9.2

Table S5 The recoveries and variation of coefficients in spiked tissue of goat

Tissue	Concentration(μg/kg)	Recoveries (%)	variation of Coefficients (%)
Liver	0.25	76.7±7.9	10.3
	0.5	75.8±5.1	6.7
	1	77.9±5.5	7.1
Kidney	0.25	76.8±5.9	7.7
	0.5	77.6±5.9	11
	1	81.3±5.5	6.8
Muscle	0.25	83.3±11	13.2
	0.5	79.5±8.1	10.2
	1	84.2±5.9	7.1
Fat	0.25	75.6±6.1	8
	0.5	74.6±7.1	9.5
	1	75.5±8.0	10.6
Lung	0.25	85.2±7.7	9.1
	0.5	83.4±6.6	7.9
	1	84.9±4.6	5.5
Large intestine	0.25	85.0±7.0	8.2
	0.5	89.3±4.0	4.5
	1	84.6±5.9	7
Small intestine	0.25	84.8±7.2	8.5
	0.5	85.4±4.1	4.7
	1	83.0±5.7	6.9
Blood	0.25	84.6±7.1	8.4
	0.5	88.2±5.1	5.8
	1	89.2±5.7	6.4
Urine	0.25	81.6±9.9	12.1
	0.5	83.0±8.0	9.6
	1	82.1±9.7	11.8
Feces	0.25	80.4±9.6	11.9
	0.5	80.1±6.9	8.6
	1	78.3±7.2	9.2
Hair	0.25	74.2±5.7	7.7
	0.5	77.8±7.4	9.5
	1	75.4±6.7	8.9

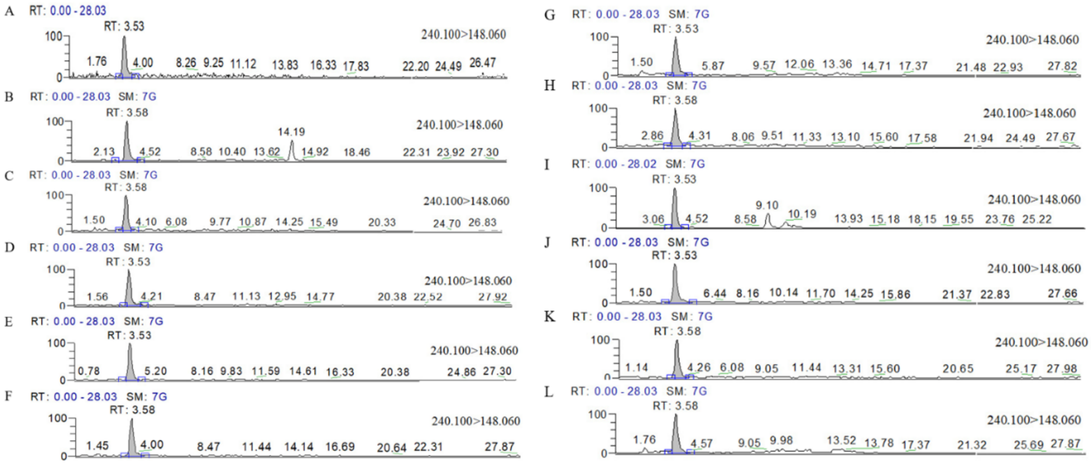

Figure S1 The chromatography of quantitative ions of salbutamol in spiked swine: (A):Blank standard solution,(B):Large intestine, (C):Liver, (D):Muscle, (E):Lung, (F):Kidney, (G):Small

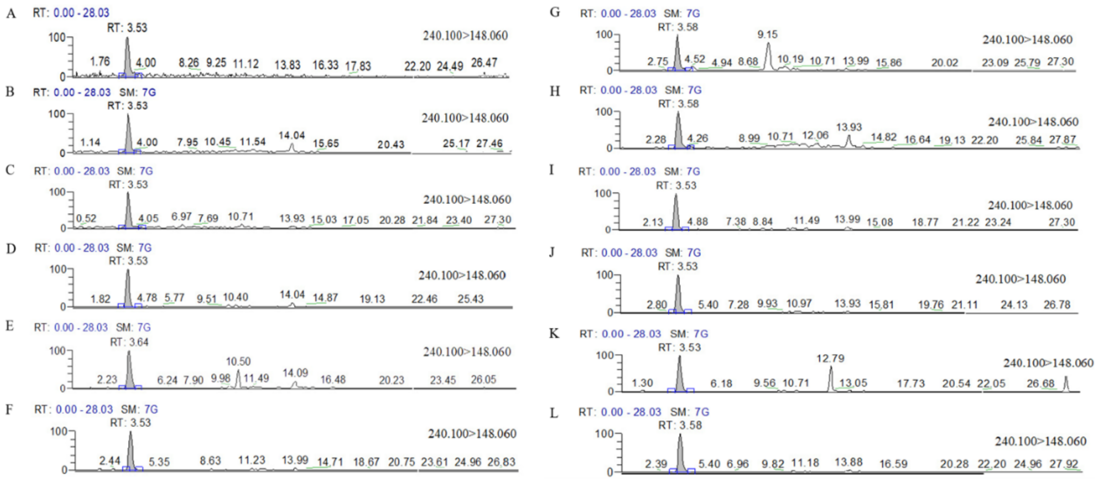

intestine,(H):Feces,(I):Hair,(J):Urine,(K):Blood,(L):Fat

Figure S2 The chromatography of quantitative ions of salbutamol in spiked goat: (A):Blank standard solution,(B):Kidney,(C): Large intestine, (D):Feces, (E):Liver, (F):Muscle, (G):Hair,(H):Urine,(I):Small intestine,(J):Blood,(K):Fat,(L):Lung

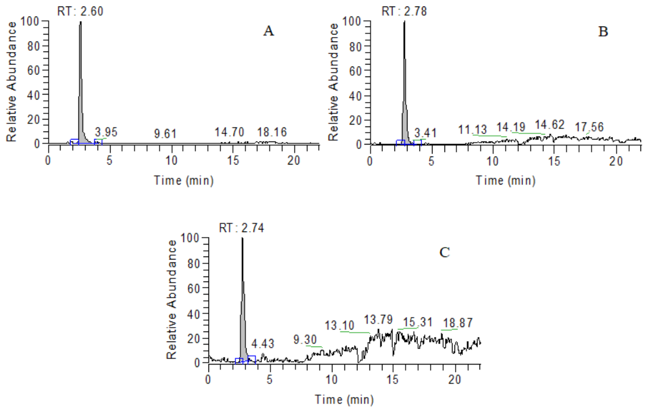

Figure S3 The chromatography of quantitative ions of Salbutamol in swine liver

((A): 6h, (B): 1d, (C): 3d)

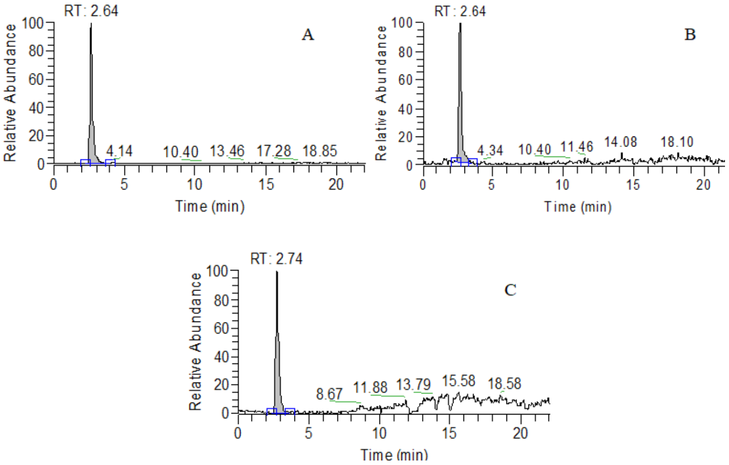

Figure S4 The chromatography of quantitative ions of Salbutamol in swine kidney

((A): 6h, (B): 1d, (C): 3d)

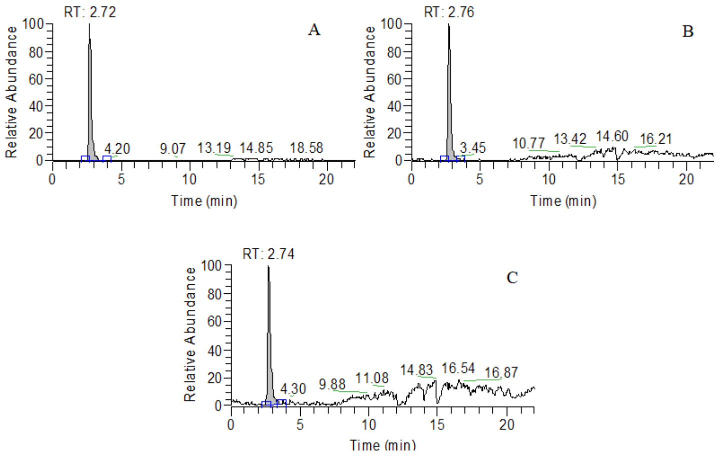

Figure S5 The chromatography of quantitative ions of Salbutamol in swine muscle

((A): 6h, (B): 1d, (C): 3d)

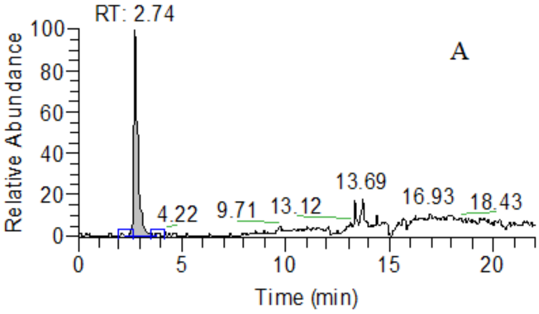

Figure S6 The chromatography of quantitative ions of Salbutamol in swine fat ((A): 6h)

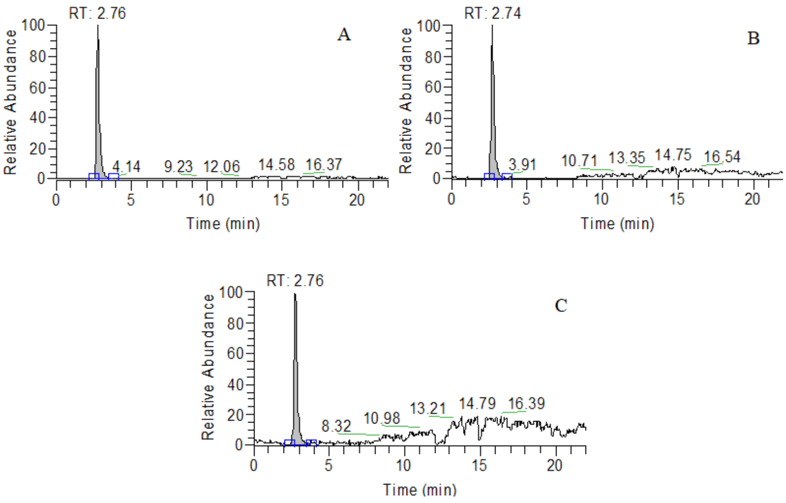

Figure S7 The chromatography of quantitative ions of Salbutamol in swine lung
((A): 6h, (B): 1d, (C): 3d)

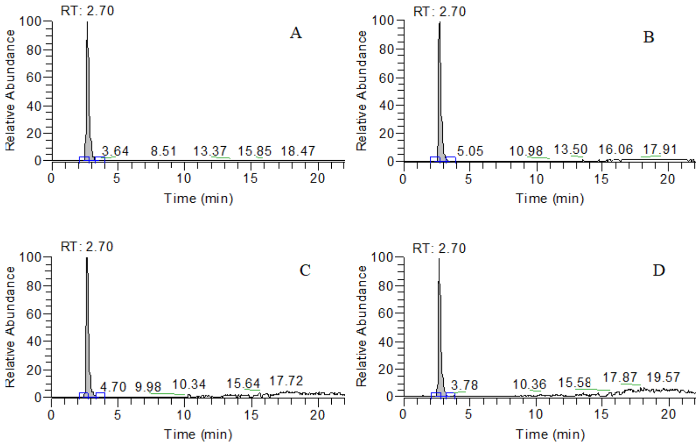

Figure S8 The chromatography of quantitative ions of Salbutamol in swine large intestine
((A): 6h, (B): 1d, (C): 3d, (D): 7d)

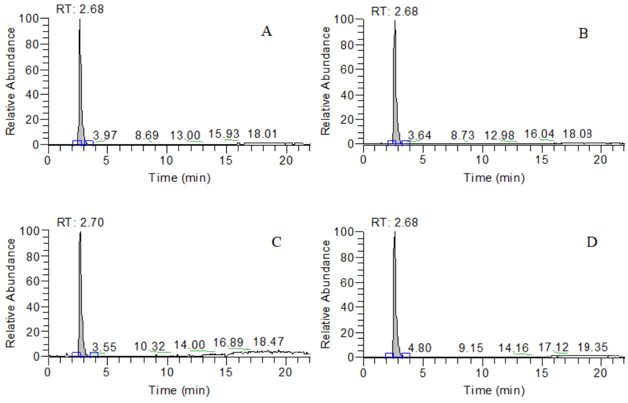

Figure S9 The chromatography of quantitative ions of Salbutamol in swine

small intestine ((A): 6h, (B): 1d, (C): 3d, (D): 7d)

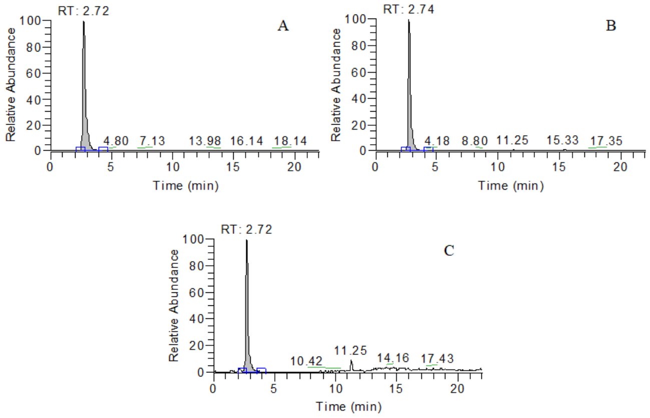

Figure S10 The chromatography of quantitative ions of Salbutamol in swine urine
((A): 6h, (B): 1d, (C): 3d)

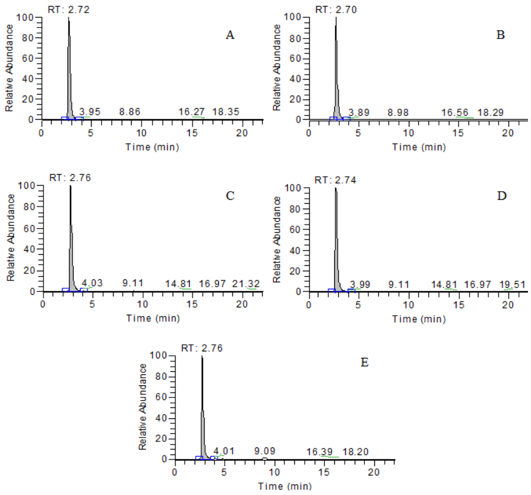

Figure S11 The chromatography of quantitative ions of Salbutamol in swine hair
((A): 6h, (B): 1d, (C): 3d, (D): 7d, (E): 14d)

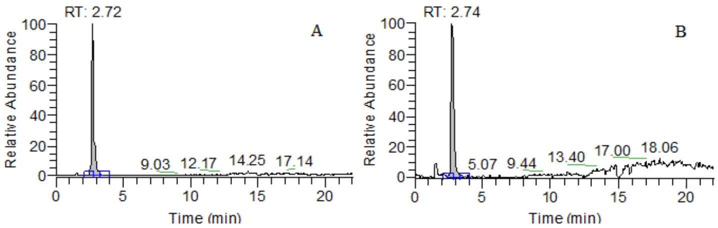

Figure S12 The chromatography of quantitative ions of Salbutamol in swine plasma
((A): 6h, (B): 1d)

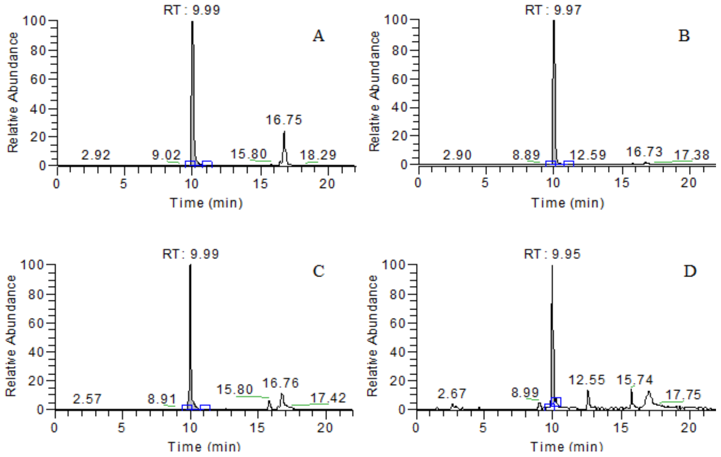

Figure S13 The chromatography of quantitative ions of Salbutamol in swine feces
((A): 6h, (B): 1d, (C): 3d, (D): 7d)

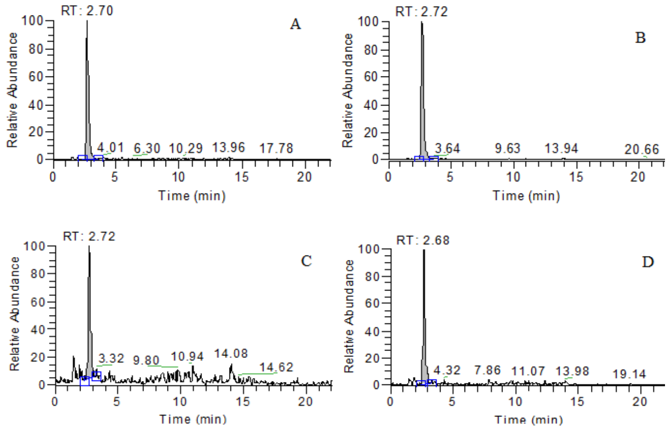

Figure S14 The chromatography of quantitative ions of Salbutamol in goat liver (enzymatic hydrolysis) ((A): 6h, (B): 1d, (C): 3d, (D): 7d)

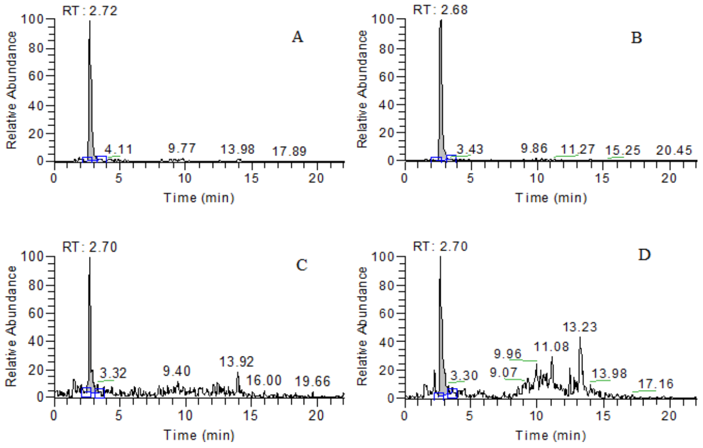

Figure S15 The chromatography of quantitative ions of Salbutamol in goat liver (without enzymatic hydrolysis) ((A): 6h, (B): 1d, (C): 3d, (D): 7d)

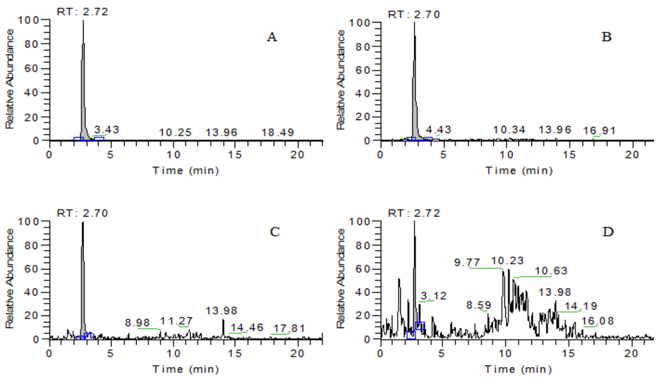

Figure S16 The chromatography of quantitative ions of Salbutamol in goat kidney (enzymatic hydrolysis) ((A): 6h, (B): 1d, (C): 3d, (D): 7d)

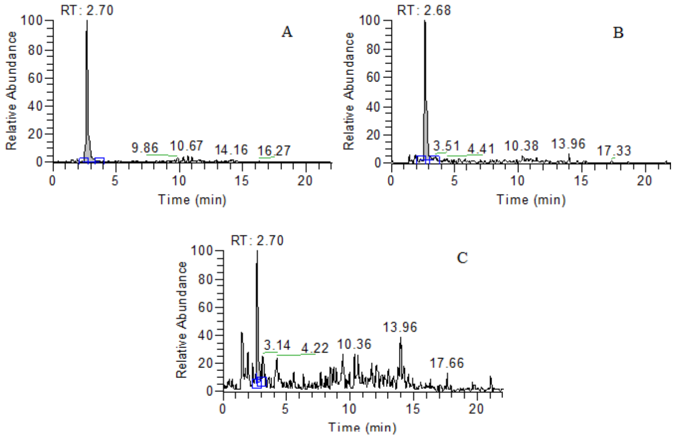

Figure S17 The chromatography of quantitative ions of Salbutamol in goat kidney (without enzymatic hydrolysis) ((A): 6h, (B): 1d, (C): 3d)

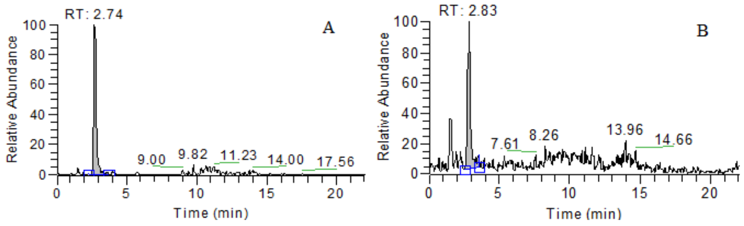

Figure S18 The chromatography of quantitative ions of Salbutamol in goat muscle (enzymatic hydrolysis) ((A): 6h, (B): 1d)

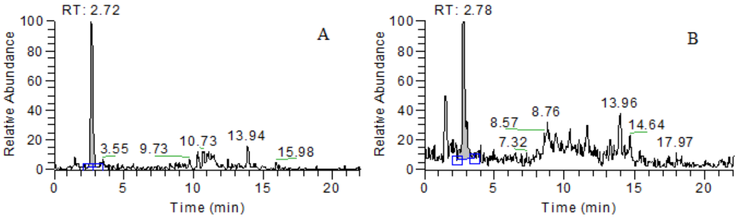

Figure S19 The chromatography of quantitative ions of Salbutamol in goat muscle (without enzymatic hydrolysis) ((A): 6h, (B): 1d)

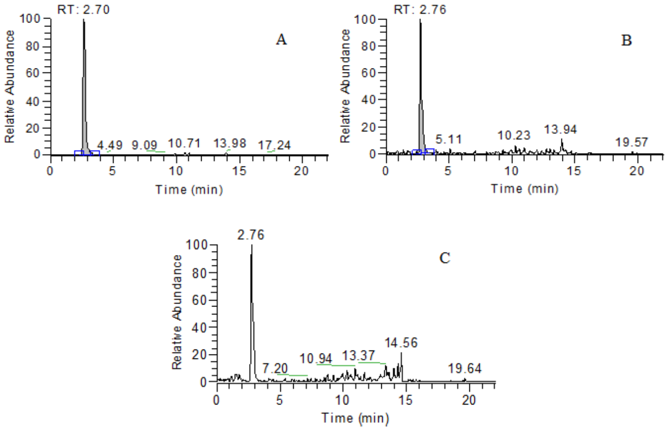

Figure S20 The chromatography of quantitative ions of Salbutamol in goat fat (enzymatic hydrolysis) ((A): 6h, (B): 1d, (C): 3d)

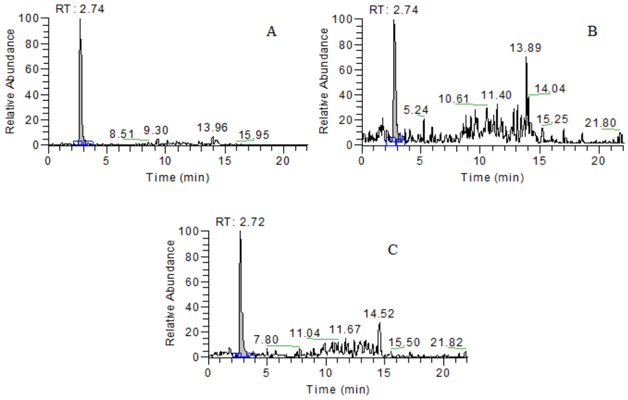

Figure S21 The chromatography of quantitative ions of Salbutamol in goat fat (without enzymatic hydrolysis) ((A): 6h, (B): 1d, (C): 3d)

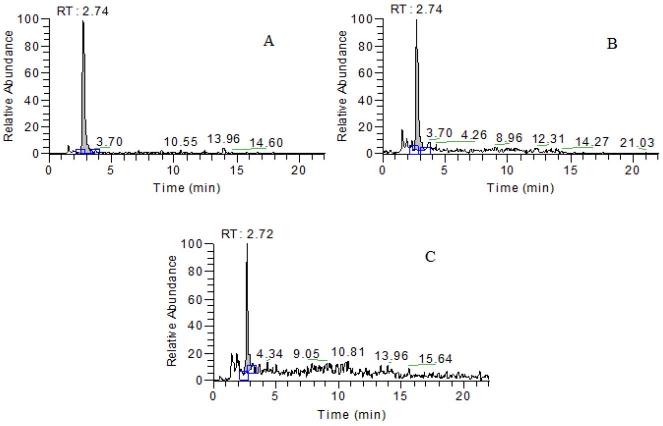

Figure S22 The chromatography of quantitative ions of Salbutamol in goat lung (enzymatic hydrolysis) ((A): 6h, (B): 1d, (C): 3d)

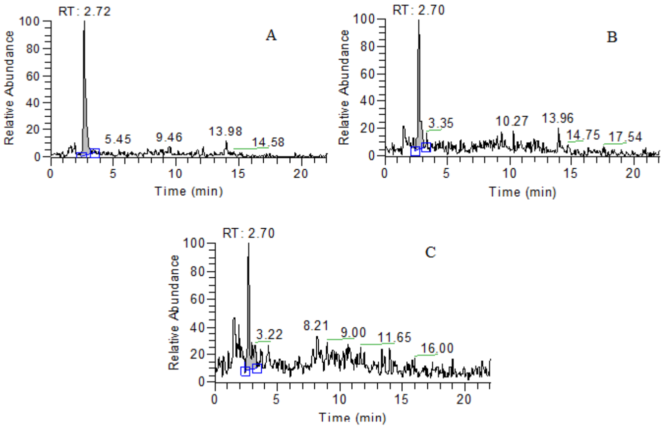

Figure S23 The chromatography of quantitative ions of Salbutamol in goat lung (without enzymatic hydrolysis) ((A): 6h, (B): 1d, (C): 3d)

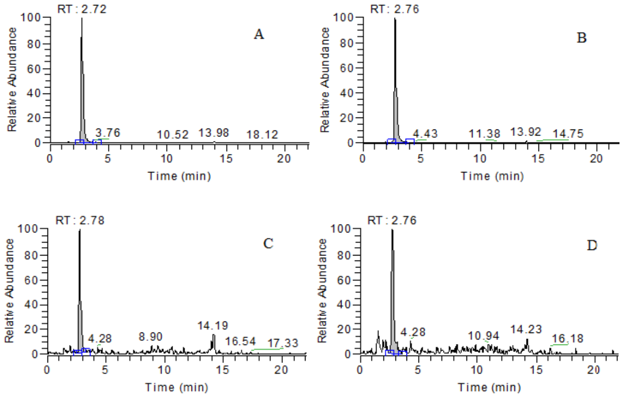

Figure S24 The chromatography of quantitative ions of Salbutamol in goat large intestine (enzymatic hydrolysis) ((A): 6h, (B): 1d, (C): 3d,(D):7d)

S

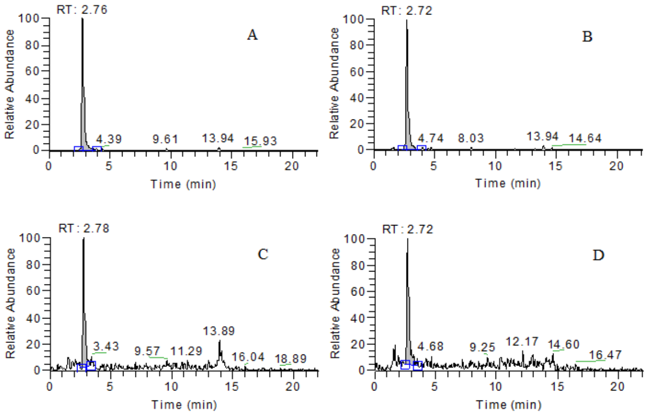

Figure S25 The chromatography of quantitative ions of Salbutamol in goat large intestine (without enzymatic hydrolysis) ((A): 6h, (B): 1d, (C): 3d, (D): 7d)

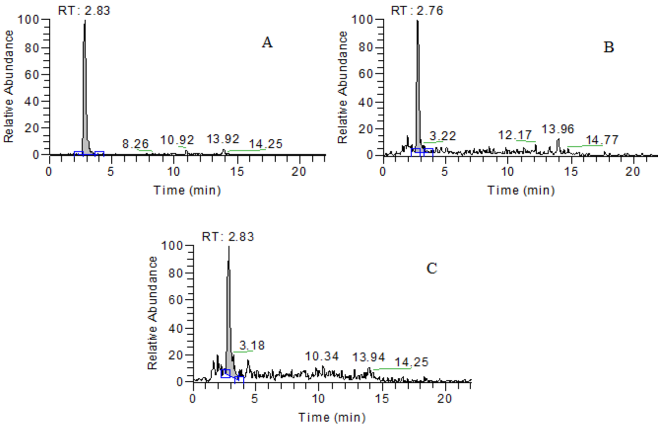

Figure S26 The chromatography of quantitative ions of Salbutamol in goat small intestine (enzymatic hydrolysis) ((A): 6h, (B): 1d, (C): 3d)

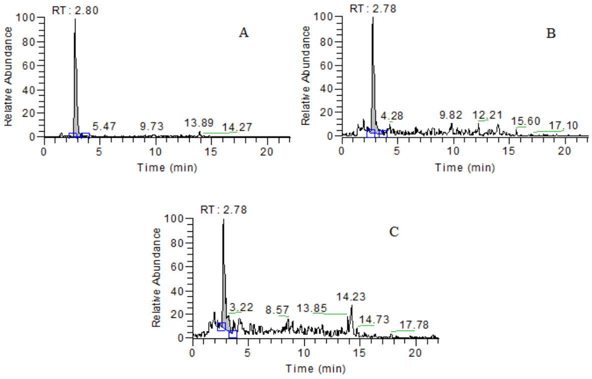

Figure S27 The chromatography of quantitative ions of Salbutamol in goat small intestine (without enzymatic hydrolysis) ((A): 6h, (B): 1d, (C): 3d)

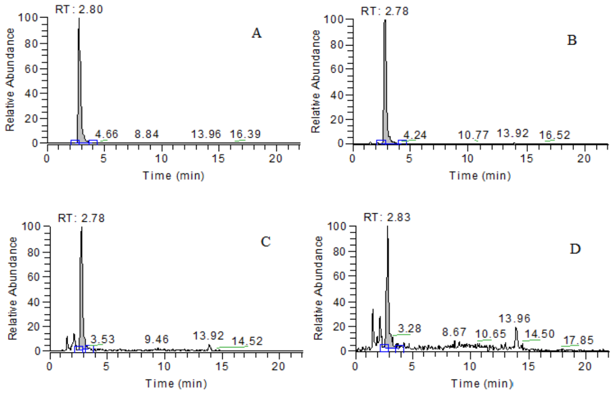

Figure S28 The chromatography of quantitative ions of Salbutamol in goat plasma (enzymatic hydrolysis) ((A): 6h, (B): 1d, (C): 3d, (D):7d)

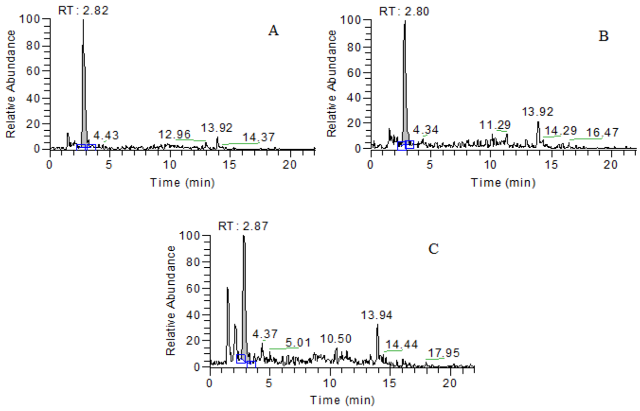

Figure S29 The chromatography of quantitative ions of Salbutamol in goat plasma (without enzymatic hydrolysis) ((A): 6h, (B): 1d, (C): 3d, (D): 7d)

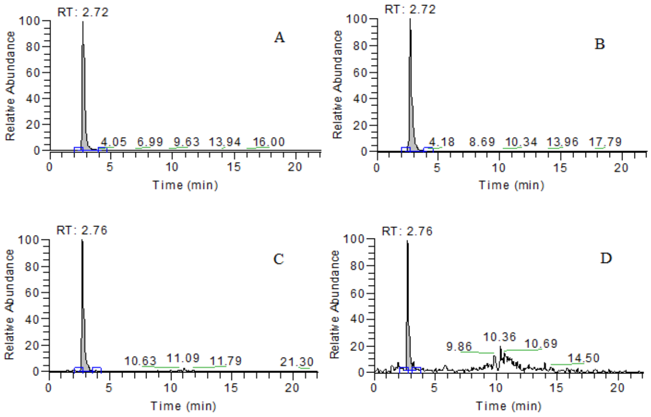

Figure S30 The chromatography of quantitative ions of Salbutamol in goat urine (enzymatic hydrolysis) ((A): 6h, (B): 1d, (C): 3d, (D): 7d)

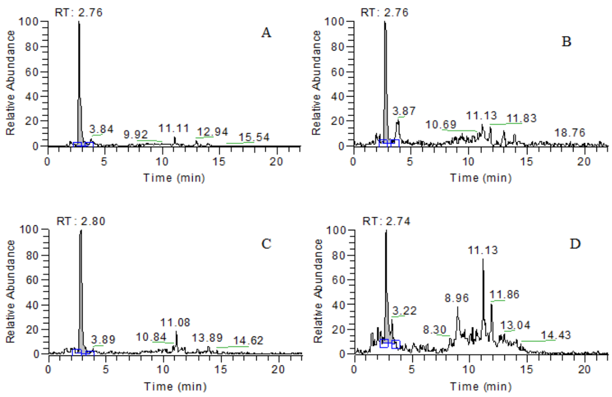

Figure S31 The chromatography of quantitative ions of Salbutamol in goat urine (without enzymatic hydrolysis) ((A): 6h, (B): 1d, (C): 3d, (D): 7d)

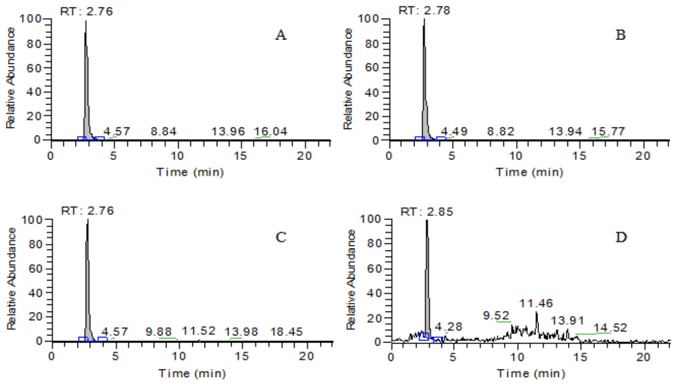

Figure S32 The chromatography of quantitative ions of Salbutamol in goat feces (without enzymatic hydrolysis) ((A): 6h, (B): 1d, (C): 3d, (D): 7d)

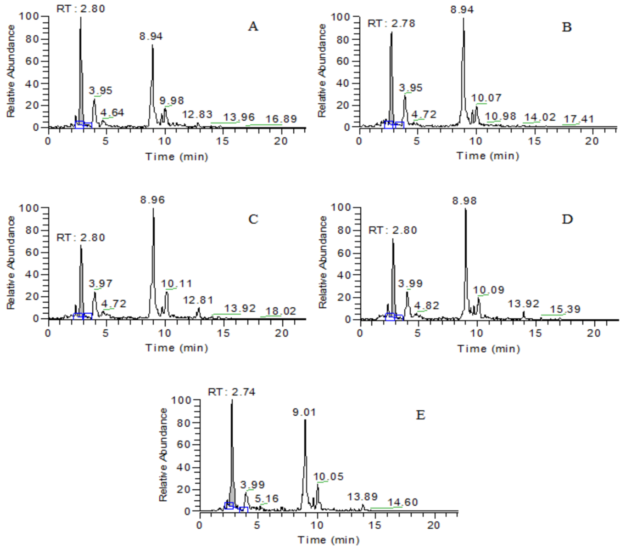

Figure S33 The chromatography of quantitative ions of Salbutamol in goat hair (without enzymatic hydrolysis) ((A): 6h, (B): 1d, (C): 3d, (D): 7d, (E): 14d)
